# The effect of population mobility on COVID-19 incidence in 314 Latin American cities: a longitudinal ecological study with mobile phone location data

**DOI:** 10.1016/S2589-7500(21)00174-6

**Published:** 2021-08-26

**Authors:** Josiah L Kephart, Xavier Delclòs-Alió, Daniel A Rodríguez, Olga L Sarmiento, Tonatiuh Barrientos-Gutiérrez, Manuel Ramirez-Zea, D Alex Quistberg, Usama Bilal, Ana V Diez Roux

**Affiliations:** Urban Health Collaborative, Dornsife School of Public Health, Drexel University, Philadelphia, PA, USA; Institute of Urban and Regional Development, University of California, Berkeley, Berkeley, CA, USA; Department of City and Regional Planning, Institute for Transportation Studies, University of California, Berkeley, Berkeley, CA, USA; School of Medicine, Universidad de Los Andes, Bogotá, Colombia; Center for Population Health Research, National Institute of Public Health of Mexico, Mexico City, Mexico; INCAP Research Center for the Prevention of Chronic Diseases, Institute of Nutrition of Central America and Panama, Guatemala City, Guatemala; Urban Health Collaborative, Department of Environmental and Occupational Health, Dornsife School of Public Health, Drexel University, Philadelphia, PA, USA; Urban Health Collaborative, Department of Epidemiology and Biostatistics, Dornsife School of Public Health, Drexel University, Philadelphia, PA, USA; Urban Health Collaborative, Department of Epidemiology and Biostatistics, Dornsife School of Public Health, Drexel University, Philadelphia, PA, USA

## Abstract

**Background:**

Little is known about the effect of changes in mobility at the subcity level on subsequent COVID-19 incidence, which is particularly relevant in Latin America, where substantial barriers prevent COVID-19 vaccine access and non-pharmaceutical interventions are essential to mitigation efforts. We aimed to examine the longitudinal associations between population mobility and COVID-19 incidence at the subcity level across a large number of Latin American cities.

**Methods:**

In this longitudinal ecological study, we compiled aggregated mobile phone location data, daily confirmed COVID-19 cases, and features of urban and social environments to analyse population mobility and COVID-19 incidence at the subcity level among cities with more than 100 000 inhabitants in Argentina, Brazil, Colombia, Guatemala, and Mexico, from March 2 to Aug 29, 2020. Spatially aggregated mobile phone data were provided by the UN Development Programme in Latin America and the Caribbean and Grandata; confirmed COVID-19 cases were from national government reports and population and socioeconomic factors were from the latest national census in each country. We used mixed-effects negative binomial regression for a time-series analysis, to examine longitudinal associations between weekly mobility changes from baseline (prepandemic week of March 2–9, 2020) and subsequent COVID-19 incidence (lagged by 1–6 weeks) at the subcity level, adjusting for urban environmental and socioeconomic factors (time-invariant educational attainment, residential overcrowding, population density [all at the subcity level], and country).

**Findings:**

We included 1031 subcity areas, representing 314 Latin American cities, in Argentina (107 subcity areas), Brazil (416), Colombia (82), Guatemala (20), and Mexico (406). In the main adjusted model, we observed an incidence rate ratio (IRR) of 2·35 (95% CI 2·12–2·60) for COVID-19 incidence per log unit increase in the mobility ratio (*vs* baseline) during the previous week. Thus, 10% lower weekly mobility was associated with 8·6% (95% CI 7·6–9·6) lower incidence of COVID-19 in the following week. This association gradually weakened as the lag between mobility and COVID-19 incidence increased and was not different from null at a 6-week lag.

**Interpretation:**

Reduced population movement within a subcity area is associated with a subsequent decrease in COVID-19 incidence among residents of that subcity area. Policies that reduce population mobility at the subcity level might be an effective COVID-19 mitigation strategy, although they should be combined with strategies that mitigate any adverse social and economic consequences of reduced mobility for the most vulnerable groups.

**Funding:**

Wellcome Trust.

## Introduction

Around 80% of the population of Latin America live in urban areas, and many of the most severe outbreaks of COVID-19 have occurred in the cities of Latin America. In attempts to mitigate the spread of COVID-19, governments have relied on regional or city-wide interventions to reduce SARS-CoV-2 transmission, by establishing universal policies such as stay-at-home restrictions. Although these widespread measures have helped to mitigate COVID-19 incidence, they have incurred substantial societal and economic costs, particularly in many cities in Latin America where widespread transmission and the associated stay-at-home restrictions have persisted.^1^

A central component of efforts to reduce COVID-19 transmission has been managing population movement, based on the intuitive idea that less population mobility leads to fewer opportunities for SARS-CoV-2 transmission. Studies that have empirically quantified the effects of mobility reductions on COVID-19 incidence have generally found positive associations between mobility and subsequent COVID-19 incidence or deaths.^2-8^ However, most of these studies were done at the country or provincial level^3,4^ or examined mobility within a single city.^6-8^ Understanding the relationship between mobility and COVID-19 incidence at the subcity level is essential to evaluate the potential effectiveness of dynamic, geographically targeted policies aimed at reducing mobility and mitigating COVID-19 incidence at a community level while minimising regional or city-wide disruption. To our knowledge, no studies have examined the effect of subnational mobility on COVID-19 incidence in the context of large and growing cities in densely urbanised low-income and middle-income countries. This gap in the research is particularly relevant in Latin America, where the pandemic has been persistent and the public health response to COVID-19 might be prolonged for many years, even as vaccine roll-out begins.^1^

To address this gap, the present analysis leverages data from anonymised mobile phone records, daily government COVID-19 reports, and national census bureaus representing 314 large, heterogeneous Latin American cities. With this rich dataset we study the association between subcity mobility changes during the first 6 months of the COVID-19 pandemic and subsequent COVID-19 incidence.

## Methods

### Study design and area

This longitudinal ecological study was done as part of the Salud Urbana en América Latina (SALURBAL; also known as Urban Health in Latin America) project. The SALURBAL project has compiled and harmonised data on health, social, and environmental characteristics for 371 cities in 11 Latin American countries.^9^ Cities were defined as urban agglomerations with more than 100 000 residents in 2010, to ensure the inclusion of a range of city sizes, from small cities to megacities. The SALURBAL project defines cities as clusters of administrative units encompassed by the visually apparent urban built up area as identified with satellite imagery.^10^ The administrative areas that compose cities are referred to as subcity areas. In this analysis, we included all subcity areas in the SALURBAL study area with the relevant COVID-19 incidence and mobility data described herein, which were in Argentina, Brazil, Colombia, Guatemala, and Mexico. Subcity areas were defined as municipios in Brazil, Colombia, Guatemala, and Mexico. In Argentina, subcity areas correspond to comunas in Buenos Aires city, partidos in Buenos Aires province, and departamentos elsewhere.^10^

### Measures and data sources

To evaluate subcity mobility, we used anonymised, spatially aggregated mobile phone data provided by the UN Development Programme in Latin America and the Caribbean and Grandata.^11^ These data include estimates of human mobility, defined as the number of trips taken away from an individual’s residence and referred to as out-of-home events, aggregated and reported at the subcity area level for mobile phone users. Mobility for a given subcity area was predefined as all out-of-home events that occurred within the subcity area, regardless of whether the mobile phone user lived within or outside the subcity area. Data were provided for each day from March 3 to Aug 29, 2020, and for a baseline pre-COVID-19 date of March 2, 2020, according to dates prespecified by the data source. These longitudinal data covered between 2% and 5% of the total population in each country.

To evaluate COVID-19 incidence at the subcity level, we used daily confirmed COVID-19 cases as reported directly at the subcity area level by the national governments of Argentina, Brazil, Colombia, Guatemala, and Mexico. COVID-19 case dates represent the date of COVID-19 diagnosis. Additional information on the compilation of COVID-19 case data by the SALURBAL project and the specific data sources are available online.

To measure socioeconomic status, we used an index of educational attainment,^12^ as this reflects both differences in formality or informality in the labour market (relating to the ability to reduce mobility, such as working from home) and differences in occupational interactions with the public and thus SARS-CoV-2 exposure. We defined residential overcrowding as the percentage of households with more than three people per room. We calculated both measures at the subcity level using data from the latest available census for each country (Argentina 2010, Brazil 2010, Colombia 2018, Guatemala 2018, Mexico 2010). We also obtained data on the total population of the subcity area, from population projections prepared by National Census Bureaus, and on population density, defined as the number of inhabitants per square km of built-up area in the subcity area. Built-up area was calculated from Facebook’s population density maps.^13^

We compiled the start and end dates of federal state-at-home restrictions for each country from the Oxford COVID-19 Government Response Tracker.^14^ In all countries, these federal restrictions remained in place after the end of the study period (Aug 29, 2020).

### Statistical analysis

We tabulated descriptive characteristics and postbaseline mobility of the subcity areas, stratified by tertiles of cumulative COVID-19 incidence per 100 000 inhabitants during the study period. Mobility estimates were originally provided as percentage change in out-of-home events on a given date compared with the baseline date (March 2, 2020), as per the formula [(postbaseline mobility/baseline mobility)−1]×100. For further descriptive assessment, we present plots of median daily percentage changes in mobility and median daily COVID-19 incidence (7-day moving averages) for subcity areas, stratified by country. To reduce the influence of day of the week on fluctuations in mobility, we calculated the weekly mean mobility for each subcity area from the week of March 2–9, 2020 (ie, prepandemic baseline week). For each subcity area, weekly mean mobility was calculated for every 7-day period following the date of the second recorded case within the subcity area (ie, week 1 was defined as the 1–7 days following the date of the second recorded case in a subcity area, week 2 as the 8–15 days following the second recorded case, and so on). We transformed the absolute percentage change in weekly mobility to the log ratio of postbaseline weekly mobility versus baseline weekly mobility during each subsequent 7-day period to the last week that included Aug 29, 2020 (range of weeks postbaseline 1–22), as per the formula ln[(postbaseline mobility change + 1)/(baseline mobility + 1)].

For the daily COVID-19 case counts, less than 1% of subcity area-days had inexplicable negative case values reported, and case counts on these days were set to zero. For each subcity area, we calculated the weekly sum of confirmed cases following the date of the second reported case, for temporal alignment with weekly mobility.

For our longitudinal study of the association between subcity area COVID-19 incidence (outcome) and subcity area mobility (in terms of change from baseline; primary exposure), we did a weekly time-series analysis beginning on the date of the second reported case in each subcity area, with 1–6-week lags between weekly mobility change and subsequent weekly cases in separate models for each lag. We adjusted for additional independent variables of time-invariant educational attainment, residential overcrowding, population density (all at the subcity level), and country, as potential confounders. We report univariable associations between each independent variable (excluding country) and the outcome (COVID-19 incidence) and full results from the adjusted model, with a 1-week lag between mobility and cases. We also report coefficients of the adjusted association between mobility and COVID-19 incidence, comparing lags of 1, 2, 3, 4, 5, and 6 weeks before COVID-19 incidence. In secondary analyses, we also explored how results for different lags varied by country. In all analyses, we used a mixed-effects negative binomial model with random intercepts for subcity area and city, with subcity area population as an offset. Coefficients and 95% CIs from the negative binomial model were exponentiated and are interpretable as incidence rate ratios (IRRs). All analyses were done in R (version 4.1.0) and modelling was done with the glmmTMB package.^15^

### Role of the funding source

The funder of the study had no role in study design, data collection, data analysis, data interpretation, or writing of the report.

## Results

Our analysis included 1031 subcity areas representing 314 cities, in Argentina (subcity areas n=107; cities n=33), Brazil (416; 151), Colombia (82; 35), Guatemala (20; 3), and Mexico (406; 92). Four subcity areas (0·4%; n=1035) within the 314 cities were excluded from the analysis due to missing mobility data. During the study period (March 2 to Aug 29, 2020), subcity areas in the analysis had a median cumulative COVID-19 incidence of 855 confirmed cases (IQR 401–1655) per 100 000 inhabitants (table 1). The median ratio for change in mobility (postbaseline weekly mobility *vs* baseline mobility) was 0·79 (IQR 0·70–0·90) for the lowest tertile of cumulative COVID-19 incidence, 0·76 (0·65–1·00) for the middle tertile, and 0·85 (0·65–1·05) for the highest tertile. Overall, the median subcity area population was 130 800 inhabitants (44 200–277 000). Based on the incidence tertiles, larger subcity area population size was associated with increased cumulative incidence of COVID-19 (table 1). Our descriptive data also indicated that COVID-19 incidence was positively associated with population density and educational attainment, and negatively associated with residential overcrowding. Subcity area characteristics and COVID-19 incidence stratified by country are presented in appendix 2 (p 1). During the study period, the median confirmed COVID-19 incidence among subcity areas was highest in Brazil (1625 confirmed cases [IQR 1078–2309] per 100 000 inhabitants) and lowest in Mexico (400 [253–600]). The median ratio of postbaseline versus baseline mobility was the highest in subcities in Brazil (0·97 [IQR 0·82–1·18]) and lowest in subcities in Argentina (0·58 [0·48–0·70).

**Figure 1:**
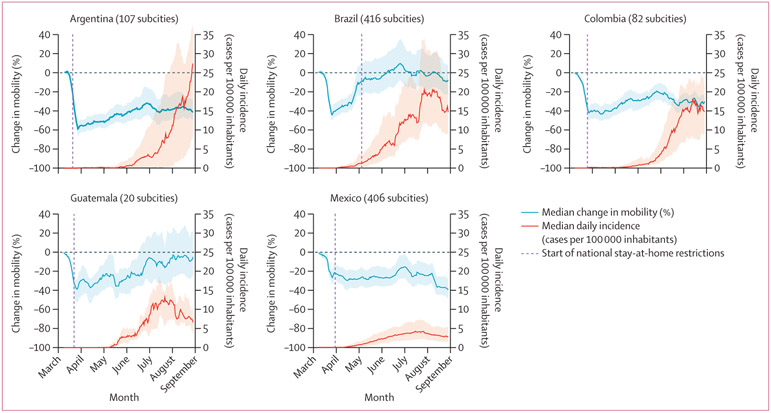
Daily change in mobility from baseline date and daily COVID-19 incidence Median values are shown as 7-day moving averages between March 2 and Aug 29, 2020. Shaded regions show the IQR of the median for all subcities. The federal stay-at-home restrictions remained in effect after the study period in all countries.

Within all five countries, subcity areas had a substantial decrease in mobility towards the end of March, as news of the pandemic spread globally and lockdown policies were implemented in many settings (figure 1). The IQRs for median percentage changes in mobility indicated wide variation between countries and between subcity areas within countries regarding the duration and patterns of the mobility reductions. Subcity areas in all countries had sharp reductions in mobility in March, 2020, even before federal restrictions began. In Argentina, Colombia, and Mexico, mobility reductions persisted in the subsequent months, with relatively narrow within-country variability. By contrast, in Brazil and Guatemala, daily mobility gradually returned to rates similar to baseline mobility, with large within-country variability.

In terms of median daily incidence of confirmed COVID-19 cases (figure 1), IQRs also showed wide within-country variation among subcity areas in the same country. By August, 2020, COVID-19 incidence in subcity areas in Brazil, Colombia, Guatemala, and Mexico was decreasing or stable, while in Argentina, COVID-19 incidence continued to increase.

We examined the univariable associations between independent variables and confirmed COVID-19 cases, and the multivariable association between mobility change from baseline (log ratio) and subsequent COVID-19 incidence (1-week lag) after adjustment (table 2). In the adjusted model, we observed an IRR of 2·35 (95% CI 2·12–2·60) for COVID-19 incidence per log unit increase in the mobility ratio during the previous week. This result indicated that a 10% lower mobility would be associated with an 8·6% (95% CI 7·6–9·6) lower COVID-19 incidence in the subsequent week, according to the equation 0·90^ln(2·35)^−1=−0·086. Interestingly, we observed a positive association between subcity area educational attainment and COVID-19 incidence (adjusted IRR 1·07 [95% CI 1·03–1·11]).

**Figure 2:**
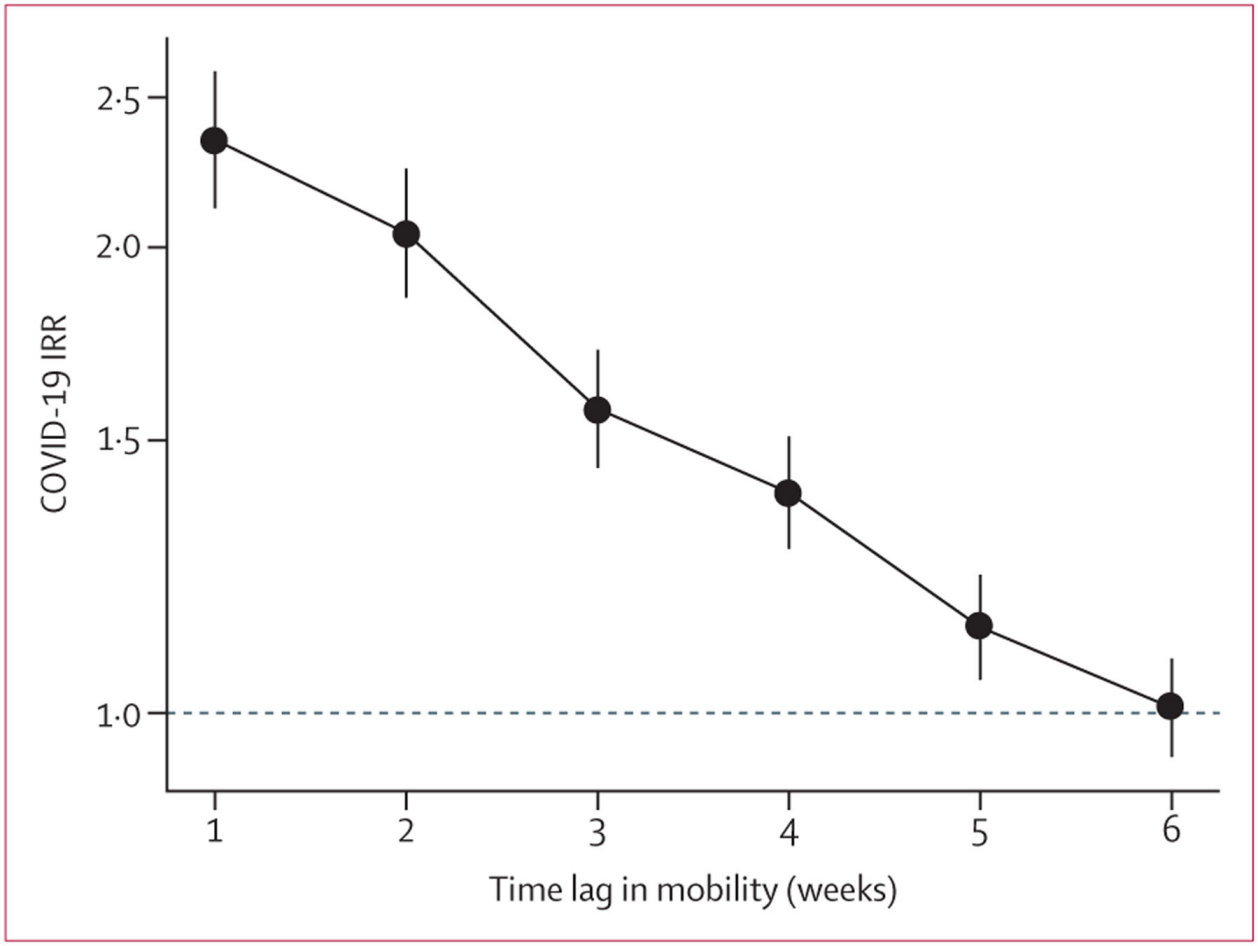
Adjusted associations between mobility change and COVID-19 incidence at the subcity level with varying time lags IRRs (per log unit increase in mobility change) are shown with 95% CIs (error bars). Adjusted models included weekly COVID-19 incidence (outcome), mobility (primary exposure), weeks since second case, population density, residential overcrowding, educational attainment, and country for 1031 subcity areas in Latin America. Weekly mobility was lagged from 1–6 weeks before weekly COVID-19 incidence in successive models. IRR=incidence rate ratio.

In analyses comparing the effects of mobility on incidence with lags of 1–6 weeks, the association between mobility and subsequent COVID-19 incidence was strongest with a lag of 1 week in the adjusted models (figure 2). This association gradually weakened from weeks 2 to 5, and for a lag of 6 weeks we observed no association between mobility and COVID-19 incidence. We also did the same analysis comparing mobility and COVID-19 incidence with 1–6-week lags stratified by country (appendix 2 p 2). Overall, declining patterns in the association between mobility and COVID-19 incidence with increasing time lag were consistent across countries. In Argentina, the association between mobility and COVID-19 incidence persisted up to 6 weeks.

## Discussion

We examined the effect of human mobility at the subcity level on subsequent COVID-19 incidence in a multicity, multicountry analysis. We observed a strong positive association between changes in population mobility within a subcity area and subsequent COVID-19 incidence among residents of that area. This association was strongest with a 1-week lag between mobility and incidence, for which a 10% lower mobility was associated with an 8·6% (95% CI 7·6–9·6) lower incidence of COVID-19. The IRR for the effect of mobility on COVID-19 incidence gradually decreased with successive weekly lags until a null effect was observed at 6 weeks. These results provide evidence that mobility is a contributor to COVID-19 incidence at the subcity level. Furthermore, the subcity level might be an effective target of interventions to reduce mobility and mitigate SARS-CoV-2 transmission, thereby potentially limiting city-wide disruption.

We observed an association between weekly mobility changes and weekly COVID-19 incidence that was strongest with a lag of 1 week, gradually weakening thereafter. Similar results were reported in a county-level analysis in the USA, which found high correlations (Pearson’s *r*>0·7) between increased COVID-19 incidence and mobility with a lag of only 4 days, with the strongest correlations at lags of 9–12 days.^2^ A provincial-level analysis in Italy found that highly affected provinces recorded decreases in COVID-19 incidence 9–10 days after mobility reductions from lockdown policies.^16^ These ranges are consistent with the reported COVID-19 latency period of a median of 5·1 days from infection to the development of symptoms.^17^ Other than time to symptom development, we would expect the average time from infection to confirmation of a COVID-19 case to vary depending on COVID-19 testing access, speed, and reporting.

We found no evidence of an association between residential overcrowding at the subcity level and COVID-19 incidence in the adjusted model. In addition, higher educational attainment was positively associated with COVID-19 incidence in the adjusted model. These findings contrast with studies in the USA showing that overcrowding at the county or zip code area level is positively associated, and education negatively associated, with COVID-19 incidence.^18,19^ However, we caution against forming conclusions from these results. Variations in testing by subcity area socioeconomic status (and overcrowding) are likely to have affected our findings, as socioeconomic status has been shown to be associated with testing.^20,21^ In addition, our study was not designed to isolate the causal associations of these variables with COVID-19 incidence as they were primarily included for adjustment purposes.^22^

Our study is strengthened by use of a rich dataset, allowing detailed analysis of 1031 subcity areas in 314 cities across five countries that have had some of the most severe COVID-19 outbreaks globally. This study also used daily mobility data compiled from anonymised mobile phones, which directly measure a community-level behaviour that contributes to SARS-CoV-2 transmission. Linking the datasets allowed longitudinal analyses that provided empirical evidence on the effectiveness of a widespread COVID-19 mitigation strategy at a fine spatial level.

This study has several limitations. Confirmed COVID-19 cases were gathered directly from official governmental sources and are likely to be under-reported, which might vary depending on access to testing and health care. However, we adjusted for subcity area education, which has been shown to be associated with access to testing.^23^ Furthermore, given our analytical approach, access to testing could only confound our results if it covaries systematically with changes in mobility with time within subcity areas. We were also limited by the scarcity of individual-level data, meaning we were unable to examine the association between individual mobility, education, and COVID-19 incidence and could not incorporate individual-level data on employment and work arrangements. The period of the study (March 2 to Aug 29, 2020) was also not long enough to fully capture any underlying seasonal changes in population mobility that might have persisted during the pandemic.

The mobility datasets from Grandata provide a metric of the total number of out-of-home events that occurred in a particular subcity area at a particular moment in time, regardless of travellers’ subcity area of residence. This means that we were unable to measure the mobility of residents of a particular subcity area, but instead measured the mobility of everyone in a particular subcity area at a given moment in time. However, this subcity level analysis remains relevant to policymakers, who might be similarly focused on regulating all mobility within specific geographical areas, rather than regulating mobility based on an individual’s location of residence. Furthermore, because the mobility data are available at the subcity level only, we were unable to examine mobility at a city level to evaluate the relative importance of city-level versus sub-city level mobility reductions in influencing COVID-19 incidence. Finally, although other non-pharmaceutical intervention, such as face-coverings, social distancing, and handwashing, might have substantial roles in mitigating community-level COVID-19 incidence, these behaviours are challenging to measure.^24^ We expect that mobility and other non-pharmaceutical interventions are correlated in timing and adherence and have distinct effects on COVID-19 incidence, which are difficult to distinguish.^5,25^ However, policies that directly regulate mobility are core actionable interventions and, in many situations, might be a first line of defence at a city or subcity level.

Geographically targeted, dynamic restrictions to encourage social distancing have been implemented at the municipal level within some metropolitan areas in Chile.^26^ Within Santiago in Chile, population movement between areas of the city has been associated with changes in COVID-19 incidence, in addition to movement within areas of the city.^8^ The geographically targeted, dynamic mobility restrictions in Chile offer valuable insight for policy makers considering localised mobility restrictions in other locations.

In this study of more than 1000 subcity areas, across 314 cities in five Latin American countries, we observed a positive association between weekly mobility changes and COVID-19 incidence. Specifically, a 10% lower weekly mobility was associated with an 8·6% lower incidence of COVID-19 the following week, and this association weakened with longer time lags. This analysis provides novel evidence from a wide range of cities with diversity in their health infrastructure, patterns of COVID-19 progression, and government responses to the pandemic. Reductions in out-of-home population movement within subcity areas decreases the risk of COVID-19 incidence among residents of those subcity areas.

Policies which aim to mitigate COVID-19 by reducing population mobility might be effective in reducing COVID-19 incidence at the subcity or neighbourhood level. However, these policies can also have substantial costs. Any mobility restrictions should be combined with policies to protect vulnerable communities, enabling them to reduce mobility and mitigating any adverse social and economic costs of reduced mobility, such as losses in income, jobs, or other sources of support. Further research is warranted to explore the effectiveness of dynamic, geographically targeted policies to reduce SARS-CoV-2 transmission and disparities on a population level.

## Supplementary Material

Supplementary material

Supplement: Spanish language abstract

## Figures and Tables

**Table 1:** Subcity area characteristics overall and stratified by cumulative COVID-19 incidence, March 2–Aug 29, 2020

	Total	Tertile of cumulative COVID-19 incidence, cases per 100 000 inhabitants		
		Lowest: ≥0 to <506·6	Middle: ≥506·6 to <1314·1	Highest: ≥1314·1 to <9403·5
Subcity areas	1031	344	343	344
Cumulative COVID-19 incidence, cases per 100 000 inhabitants	855 (401–1655)	307 (183–401)	855 (664–1081)	2023 (1656–2602)
Change in mobility, ratio of postbaseline *vs* baseline weekly mobility*	0·80 (0·67–0·99)	0·79 (0·70–0·90)	0·76 (0·65–1·00)	0·85 (0·65–1·05)
Population, thousands	130·8 (44·2–277·0)	83·6 (28·4–181·5)	136·3 (46·3–304·1)	176·6 (82·7–345·4)
Population density, population (in thousands) per square km	8·2 (5·5–14·0)	7·3 (5·5–11·4)	9·0 (5·8–15·5)	8·1 (5·2–15·3)
Residential overcrowding, %†	5·3 (2·8–10·9)	10·9 (6·7–14·7)	5·2 (2·8–9·8)	3·1 (1·7–5·0)
Population educational attainment index‡	−0·84 (−1·60 to −0·02)	−1·38 (−2·12 to −0·56)	−0·66 (−1·35 to 0·14)	−0·56 (−1·18 to 0·26)

Data are n or median (IQR).

* Baseline mobility for March 2–9, 2020.

† Proportion of households with more than three people per room.

‡ Index that includes an average of the Z scores of the population (%) aged 25 years or older in the subcity area who have completed secondary education or higher, and the population (%) aged 25 years or older who have completed university education or higher.^12^

**Table 2:** Associations between subcity area COVID-19 weekly incidence (outcome), weekly mobility (primary exposure), and subcity area characteristics from mixed-effects negative binomial models

	Unadjusted*		Adjusted†	
	IRR (95% CI)	p value	IRR (95% CI)	p value
Mobility, ratio of change from baseline (per log unit increase)‡	8·12 (6·95–9·49)	<0·0001	2·35 (2·12–2·60)	<0·0001
Weeks since second case (per 1 week increase)	1·18 (1·17–1·18)	<0·0001	1·17 (1·16–1·17)	<0·0001
Population density (per 1000 people per square km increase)	1·01 (1·00–1·01)	<0·0001	1·00 (0·99–1·01)	0·10
Residential overcrowding (per 1% increase in overcrowded households)	0·96 (0·95–0·96)	<0·0001	0·99 (0·98–1·00)	0·23
Population educational attainment (per 1 unit increase in educational attainment index)	1·12 (1·10–1·15)	<0·0001	1·07 (1·03–1·11)	0·0011

IRR=incidence rate ratio.

* Unadjusted models include weekly COVID-19 incidence (outcome) and each single variable (exposure) for 1031 subcity areas in Latin America.

† Adjusted models include weekly COVID-19 incidence (outcome), change in mobility (primary exposure), weeks since second case, population density, residential overcrowding, population educational attainment (all on the subcity level), and country.

‡ 1-week lag between mobility and incidence.

## Research in context

### Evidence before this study

We searched PubMed and Google Scholar for articles in any language published from Jan 1, 2020 up to May 20, 2021, using the search terms “COVID-19,” “incidence,” “cases,” “mobility,” and “population movement.” We found a collection of articles that had used mobility metrics derived from anonymised mobile phone records to explore linkages to COVID-19 incidence. Most of these analyses were done at the country, provincial, or county level. Notable exceptions were analyses that examined intracity mobility in single city analyses of New York City (NY, USA), Hong Kong (China), and Santiago (Chile). The existing literature generally supports a positive association between population mobility and COVID-19 incidence at the national, regional, and county levels. However, evidence on the association between mobility and COVID-19 incidence in the large and growing urban areas of low-income and middle-income countries is sparse. As the pandemic escalates in many countries of the global south, it is important to document the potential effect of various strategies, including targeted mobility restrictions, on the evolution of the pandemic in these contexts.

### Added value of this study

This study reports longitudinal associations between population mobility and COVID-19 incidence at the subcity level in 314 heterogeneous cities across Latin America, which is one of the global regions currently most affected by the COVID-19 pandemic. To our knowledge, this study is the first to examine the effect of subnational mobility on COVID-19 incidence in a multicity study in Latin America, and one of the first studies in any location to examine this effect at the subcity level among a large number of cities in low-income and middle-income countries. We found that 10% lower weekly population mobility was associated with an 8·6% lower weekly COVID-19 incidence during the following week at a subcity level.

### Implications of all the available evidence

The existing literature suggests that high levels of international and intercity mobility lead to severe COVID-19 outbreaks. Our study provides evidence of this association at the subcity level and within Latin America. The findings contribute evidence that interventions to promote social distancing that target specific areas within cities might substantially mitigate SARS-CoV-2 transmission while reducing regional or citywide disruption.
