# Supplementary material for: The effect of population mobility on COVID-19 incidence in 314 Latin American cities: a longitudinal ecological study with mobile phone location data
Source: Lancet Digit Health. Author manuscript; Available in PMC 2021 Nov 1. (PMC8545654; doi:10.1016/S2589-7500(21)00174-6)
Supplement: Supplementary file 1 [file EMS134936-supplement-Supplementary_file_1.pdf]

THE LANCET
Digital Health

Supplementary appendix

This translation in Spanish was submitted by the authors and we reproduce it as supplied. It has not been peer reviewed. *The Lancet*'s editorial processes have only been applied to the original in English, which should serve as reference for this manuscript.

Los autores nos proporcionaron esta traducción al español y la reproducimos tal como nos fue entregada. No la hemos revisado. Los procesos editoriales de *The Lancet* se han aplicado únicamente al original en inglés, que debe servir de referencia para este manuscrito.

Supplement to: Kephart JL, Delclòs-Alió X, Rodríguez DA, et al. The effect of population mobility on COVID-19 incidence in 314 Latin American cities: a longitudinal ecological study with mobile phone location data. *Lancet Digit Health* 2021; published online Aug 26. https://doi.org/10.1016/S2589-7500(21)00174-6.

Impacto de la movilidad de la población en la incidencia de COVID-19 en 314 ciudades Latinoamericanas: un estudio ecológico longitudinal

Resumen

Antecedentes: Existen pocas evidencias sobre el impacto de los niveles de movilidad de la población a nivel de sub-ciudad en la incidencia de la COVID-19.

Métodos: Recopilamos datos agregados de la ubicación de teléfonos móviles, de casos confirmados de COVID-19 y de características del entorno urbano y social para realizar un análisis ecológico longitudinal de la relación entre la movilidad de la población y la incidencia de COVID-19. Realizamos un análisis a nivel de sub-ciudad en ciudades de más 100.000 habitantes en Argentina, Brasil, Colombia, Guatemala y México, desde el 2 de marzo al 29 de agosto de 2020. Utilizamos una regresión binomial negativa de efectos mixtos para examinar las asociaciones longitudinales entre los cambios en la movilidad semanal de la población (con retrasos temporales de 1 a 6 semanas) y la incidencia de COVID-19 a nivel de sub-ciudad, ajustando por factores ambientales y socioeconómicos.

Hallazgos: En las 1.031 áreas analizadas a nivel de sub-ciudad, localizadas en 314 ciudades de cinco países de América Latina, observamos como una reducción del 10% en los niveles de movilidad semanal se asoció con una reducción del 8,6% (IC del 95%: 7,6% a 9,6%) en la incidencia de COVID-19 en la siguiente semana. Esta asociación decayó gradualmente a medida que aumentaba el desfase temporal entre los niveles de movilidad y la incidencia de COVID-19, hasta no ser distinta del resultado nulo a las seis semanas.

Interpretación: Una menor movilidad de la población a nivel de sub-ciudad se asocia con una menor incidencia posterior de COVID-19 entre los residentes de esa área. Las políticas dirigidas a reducir la movilidad de la población a nivel de sub-ciudad pueden ser consideradas estrategias efectivas para la mitigación de la COVID-19, aunque deben combinarse con medidas dirigidas a mitigar cualquier consecuencia social y económica adversa que resulte de una reducción en la movilidad entre aquellos colectivos más vulnerables.

Palabras clave: SARS-CoV-2, movilidad, COVID-19, movimiento poblacional, confinamiento, América Latina
