# Supplementary material for: The effect of population mobility on COVID-19 incidence in 314 Latin American cities: a longitudinal ecological study with mobile phone location data
Source: Lancet Digit Health. Author manuscript; Available in PMC 2021 Nov 1. (PMC8545654; doi:10.1016/S2589-7500(21)00174-6)
Supplement: Supplementary file 2 [file EMS134936-supplement-Supplementary_file_2.pdf]

THE LANCET
Digital Health

Supplementary appendix

This appendix formed part of the original submission and has been peer reviewed. We post it as supplied by the authors.

Supplement to: Kephart JL, Delclòs-Alió X, Rodríguez DA, et al. The effect of population mobility on COVID-19 incidence in 314 Latin American cities: a longitudinal ecological study with mobile phone location data. *Lancet Digit Health* 2021; published online Aug 26. https://doi.org/10.1016/S2589-7500(21)00174-6.

Table S1. Sub-city area characteristics among 1,031 sub-city areas in 314 Latin American cities overall and stratified by country

	Total	Argentina	Brazil	Colombia	Guatemala	Mexico
Sub-city areas (n)	1,031	107	416	82	20	406
Cities represented by sub-city areas (n)	314	33	151	35	3	92
Cumulative COVID-19 cases per 100k inh. [a]	855 [401, 1,655]	959 [162, 1775]	1,625 [1078, 2309]	1,000 [574, 1474]	848 [553, 1167]	400 [253, 600]
Change in mobility (ratio) [a, c]	0.80 [0.67, 0.99]	0.58 [0.48, 0.70]	0.97 [0.82, 1.18]	0.71 [0.62, 0.76]	0.86 [0.67, 1.22]	0.75 [0.66, 0.85]
Population (thousands) [a]	130.8 [44.2, 277.0]	209.7 [133.4, 350.7]	138.0 [61.1, 273.2]	143.0 [80.0, 310.8]	109.3 [44.1, 171.3]	82.5 [25.5, 237.72]
Pop. density (pop. in thousands / sq.km) [a]	8.2 [5.5, 14.0]	8.1 [5.3, 11.8]	6.6 [4.7, 10.6]	16.7 [11.6, 24.0]	14.6 [10.4, 18.0]	8.7 [6.2, 13.4]
Residential overcrowding (%) [a, d]	5.3 [2.8, 10.9]	3.8 [2.4, 5.2]	2.7 [1.7, 4.1]	5.1 [3.3, 7.3]	22.2 [16.6, 27.5]	11.2 [8.1, 14.9]
Population educational attainment [a, e]	-0.84 [-1.60, -0.02]	-0.59 [-1.34, 0.02]	-0.61 [-1.35, 0.07]	-0.69 [-1.25, -0.02]	-2.08 [-2.68, -0.77]	-1.19 [-1.98, -0.16]

[a] *Values correspond to medians [Q1, Q3].*
[c] *Ratio of overall vs baseline mobility (March 2nd-9th, 2020).*
[d] *Proportion of households with more than 3 people per room.*
[e] *Index that includes an average of the z-scores of the % population in the sub-city area aged 25 or above that has completed secondary education or above, and the % population aged 25 or above that has completed university education or above.*

Figure S1. Incidence rate ratios (IRR) and 95% confidence intervals of the adjusted association between lagged mobility change and COVID-19 incidence at the sub-city level stratified by country, comparing mobility from one to six weeks prior to COVID-19 incidence.[a]

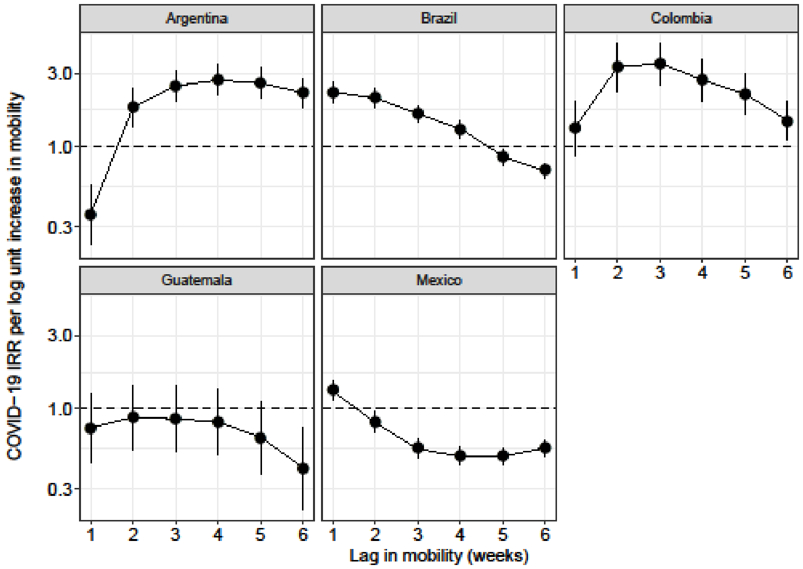

[a] Results come from adjusted models that include sub-city area weekly COVID-19 incidence (outcome), mobility (primary exposure), weeks since 2nd case, population density, residential overcrowding, educational attainment, and country for 1,031 sub-city areas in Latin America. Weekly mobility was lagged from one to six weeks prior to weekly COVID-19 incidence in subsequent models.
